# Supplementary material for: An Inferred Ancestral CotA Laccase with Improved Expression and Kinetic Efficiency
Source: Int J Mol Sci. 2023 Jun 30;24(13):10901. doi: 10.3390/ijms241310901 (PMC10341886; doi:10.3390/ijms241310901)
Supplement: Supplementary file 1 [file ijms-24-10901-s001.zip › ijms-2437522-supplementary.pdf]

Supplementary Material

>BscotA
MTLEKFVDALPIPDTLKPVQQSKEKTYYEVTMEECTHQLHRDLPPTRLWGYN
GLFPGPTIEVKRNENVYVKWMNNLPSTHFLPIDHTIHHSDSQHEEPEVKTVVH
LHGGVTPDDSDGYPEAWFSKDFEQTGPYFKREVYHYPNQQRGAILWYHDHA
MALTRLNVYAGLVGAYIIHDPKEKRLKLPSDEYDVPLLITDRTINEDGSLFYPS
APENPSPSLPNPSIVPAFCGETILVNGKVWPYLEVEPRKYRFRVINASNTRTYN
LSLDNGGDFIQIGSDGGLLPRSVKLNSFSLAPAERYDIIIDFTAYEGESIILANSA
GCGGDVNPETDANIMQFRVTKPLAQKDESRKPKYLASYPSVQHERIQNIRTLK
LAGTQDEYGRPVLLLNNKRWHDPVTETPKVGTTEIWSIINPTRGTHPIHLHLV
SFRVLDRRPFDIARYQESGELSYTGPAVPPPPSEKGWKDTIQAHAGEVLRIAAT
FGPYSGRYVWHCHILEHEDYDMMRPMDITDPHK
>AncCotA1
MTLEKFVDALPIPDTLKPVQQSKESTYYEVTMEECYHQLHRDLPPTRLWGYN
GLFPGPTIEVKRNENVYVKWMNNLPSKHFLPIDHTIHHSDSQHEEPEVKTVVH
LHGGVTPDDSDGYPEAWFSKDFEQTGPYFKREVYHYPNQQRGAILWYHDHA
MALTRLNVYAGLVGAYIIHDPKEKRLKLPSGEYDVPLLITDRTINEDGSLFYPS
GPENPSPSLPNPSIVPAFCGDTILVNGKAWPYMEVEPRKYRFRVINASNTRTYN
LSLDNGGEFIQIGSDGGLLPRSVKLNSFSLAPAERFDIIIDFAAFEGQSIILANSA
GCGGDVNPETDANIMQFRVTKPLAQKDESRKPKYLASYPSVQNERIQNIRTLK
LAGTQDEYGRPVLLLNNKRWHDPVTEAPKVGTTEIWSIINPTRGTHPIHLHLV
SFRVLDRRPFDTARYQESGELAYTGPAVPPPPSEKGWKDTVQSHAGEVLRIAA
TFGPYSGRYVWHCHILEHEDYDMMRPMDITDPHK
>AncCotA2
MNLEKFVDELPIPETLKPVKQSKESTYYEVTMKECYHKLHRDLPPTRLWGYN
GLFPGPTIEVNRNENVYVKWMNDLPDKHFLPVDHTIHHSESHHQEPEVKTVV
HLHGGVTPPDSDGYPEAWFTRDFEETGPYFKREVYHYPNQQRGAILWYHDH
AMALTRLNVYAGLAGMYIIRDKKEKRLKLPAGEYDVPLMIMDRTLNEDGSLF
YPSGPDNPSPTLPNPSIVPAFCGDTILVNGKAWPYMEVEPRKYRFRILNASNTR
TYNLSLDNGGEFIQIGSDGGLLPRSVKLQSISLAPAERFDVIIDFSAFEGQSIILT
NSAGCGGDVNPETDANVMQFRVTKPLKGKDTSRKPKYLSSLPSVTSERIQNIR
TLKLTGTQDEYGRPVLLLNNKRWHDPVTEAPKLGTTEIWSIINPTRGTHPIHLH
LVSFRVLDRRPFDTERYNETGEIVYTGPAVPPPPSEKGWKDTVQAHAGEVIRIA
ATFGPYSGRYVWHCHILEHEDYDMMRPMDVTDKQQ
>AncCotA3
MNLEKFVDELPIPETLKPVKKNKKSTYYEVTMKECYQKLHRDLPPTRLWGY
NGMFPGPTIEVNRNENVYVKWMNDLPDKHFLPVDHTIHHGESHHQEPEVKT
VVHLHGGVTPPDSDGYPEAWFTRDFEETGPYFKREVYQYPNHQRAATLWYH
DHAMALTRLNVYAGLAGYIIRDKKEKSLKLPKGEYDIPLMIMDRTFNEDGSLF
YPSQPDNPSPNLPNPSIVPAFCGDTILVNGKVWPYLEVEPRKYRFRILNASNTR
TYELSLDNGASFIQIGSDGGFLPRPVKLQSISLAPAERFDVIIDFSAYEGQTIILK
NSSGCGGDVNPETDANVMQFRVTRPLKGKDTSRIPKYLSSLPSLTQSRVQRIRT
LKLTGTQDEYGRPVLLLDNKMWHDPVTEKPRLGSTEIWSIINPTRGTHPIHLH

LVQFRVLDRRPFDTDLYNETGEIVYTGPAVPPPPSEKGWKDTVQAHAGEVIRII
ARFGPYSGRYVWHCHILEHEDYDMMRPMDVIKKKQ
>AncCotA4
MKLEKFVDPLPIPETLKPVRKNKKSTYYEVTMKEFRQKLHRDLPPTRLWGYN
GMFPGPTIEVQRNELVHVKWMNDLPDKHFLPVDKTIHHVEMHAHNPEVRTV
VHLHGGETPPDSDGYPEAWFTKDFKEVGPYFKREVYEYPNHQRAATLWYHD
HAMGITRLNVYAGLAGFYIIRDKHEKSLNLPKGEYEIPLMIQDRSFNEDGSLFY
PSQPDNPSPNLPNPSIVPAFCGDTILVNGKVWPYLEVEPRKYRFRILNASNTRS
YELSLDSGQPFYQIGTDGGLLQKPVKIEKITLAPAERVDVIIDFSKYEGQTIILK
NDSGCGGPVDPETTANVMQFRVTLPLSGKDTSRIPKYLSSIPSLSQNSVQRIRN
LKLTGSTDEYGRPLLLLDNKMWHDPVTEKPRLGDTEIWSLINVTGFTHPIHLH
LVQFQILDRRPFDVDLYNETGQIVFTGPAVPPEPNERGWKDTVRAPAGQVTRII
ARFGPYTGRYVWHCHILEHEDYDMMR
PFEVIKKKQ
>AncCotA5
MKLEKFVDPLPIPETLKPVRKNKKSTYYEVTMKEFKQKLHRDLPPTRLWGYE
GMFPGPTIEVQRNELVHVKWMNDLPDKHFLPVDKTIHHVEMEANNPEVRTV
VHLHGGETPPDSDGYPEAWFTKDFKETGPYFTREVYQYPNHQRAATLWYHD
HAMGITRLNVYAGLAGFYLIRDKHEKSLNLPKGEYEIPLMIQDRSFNEDGSLF
YPSQPDPPSPNLPNPSIVPAFCGDTILVNGKVWPYLEVEPRKYRFRILNASNTRS
YTLSLDSGQPFYQIGTDGGLLQKPVKVEKITLAPAERVDVIIDFSKYEGQTIILK
NDSGCGGPVDPETTANVMQFRVTLPLSSKDTSRIPKYLSSIPSLSQNSVQRIRN
LKLTGSTDEYGRPLLLLDNKMWHDPVTEKPRLGDTEIWSLINVTAFTHPIHLH
LVQFQILDRQPFDVDHYNETGQIVFTGPAVPPEPNERGWKDTVRAPPGQVTRII
ARFGPYTGRYVWHCHILEHEDYDMMRPFEVIKKKQ

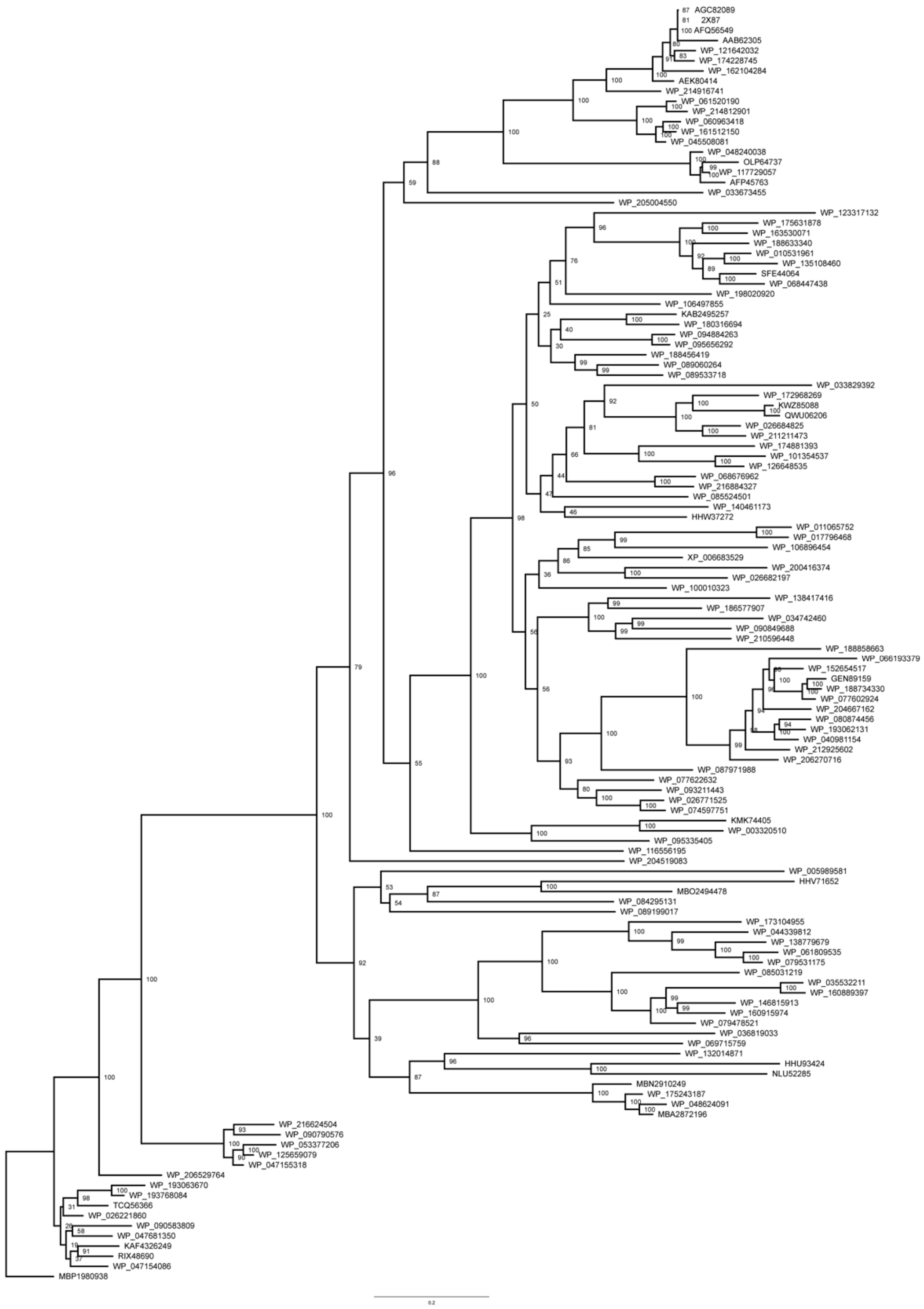

Supplementary Figure S1. A phylogenetic analysis of the BsCotA-like laccases. An unrooted Maximum-likelihood tree was constructed using IQ-Tree showing the phylogenetic relationship of BsCotA-like laccases. The confidence level of each node was estimated by the bootstrap procedure using 10,000 resampling repetitions of the data. Bootstrap values (% re-sampled data set) are indicated for each node. The multiple sequences used for the alignment and to generate the tree are available in Supplemental Dataset S1.

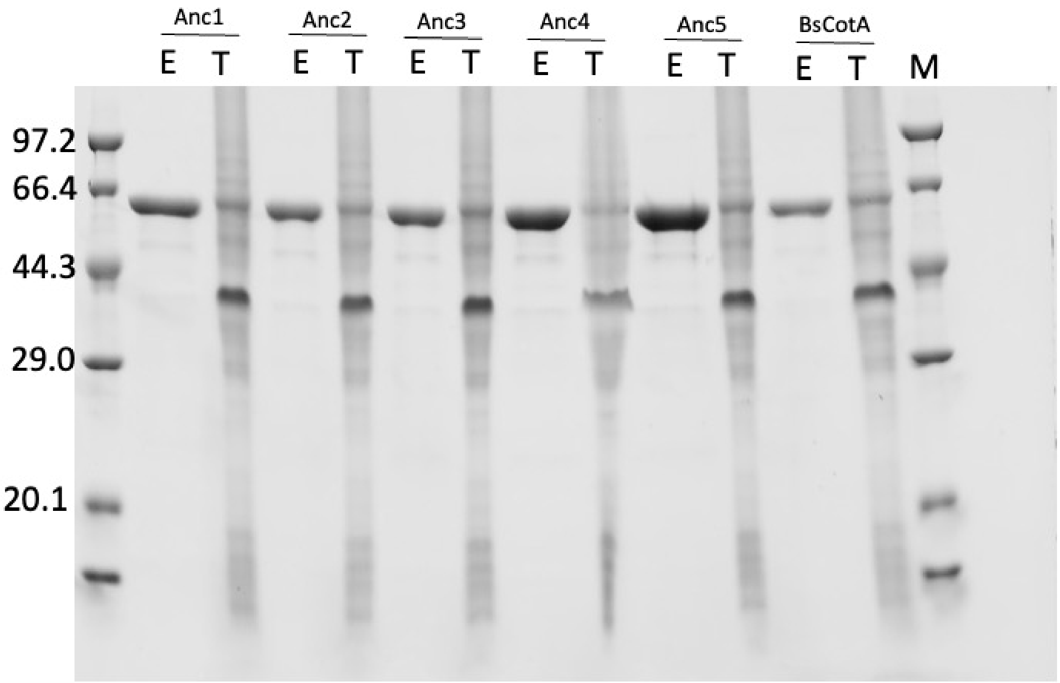

Supplementary Figure S2. SDS-PAGE analysis of laccase BsCotA and CotA Ancestors. The SDS-PAGE shows crude bacterial lysates and purified enzyme samples (T—supernatant, E—eluted fraction).

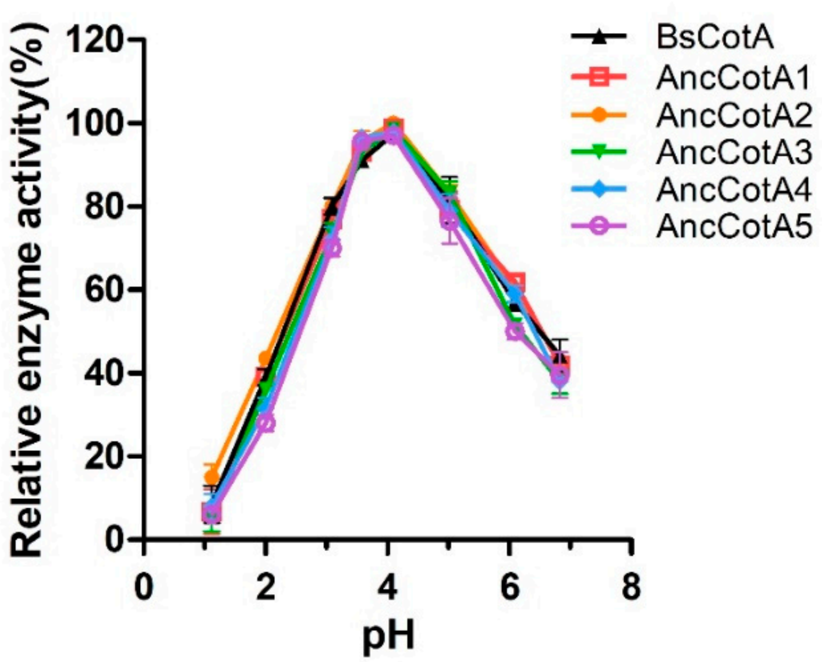

Supplementary Figure S3. Optimum pH for five designs AncCotA and BsCotA.

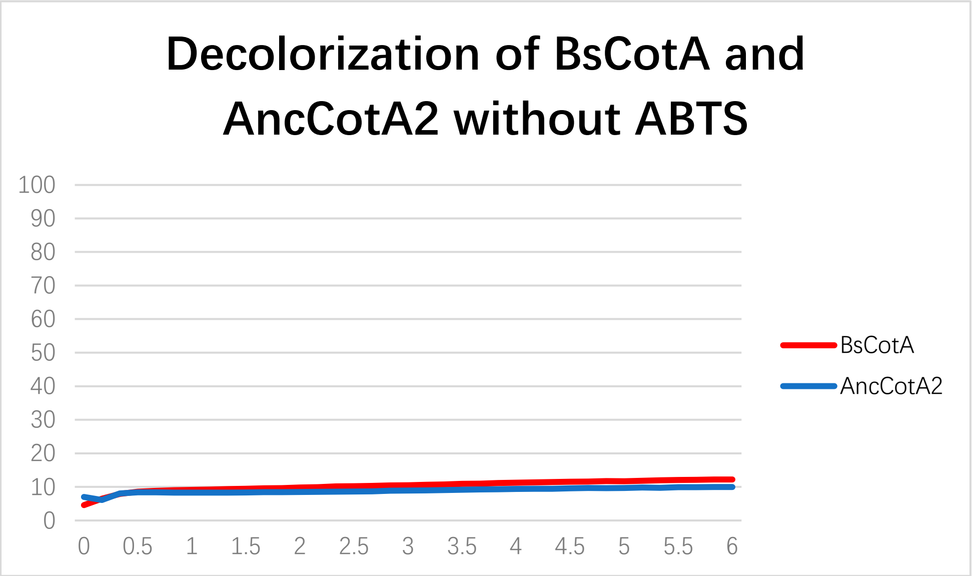

Supplementary Figure S4. Decolorization rate of 0.1 mg/mL indigo carmine by BsCotA and AncCotA2 at 3.33 μg/mL without ABTS in 6 h.

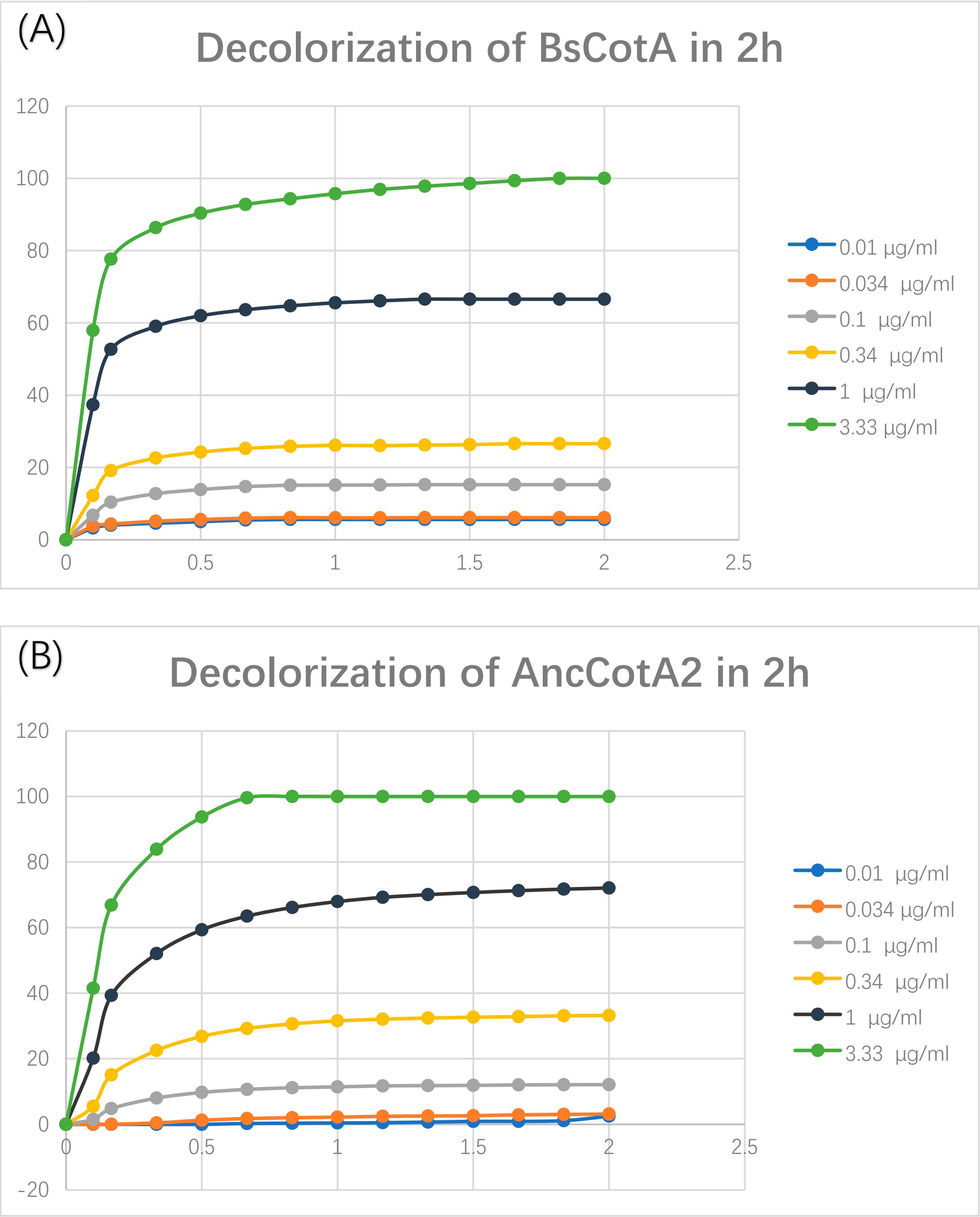

Supplementary Figure S5. (**A**) Decolorization rate of 0.1 mg/mL indigo carmine by BsCotA at different concentrations within 2 h. (**B**) Decolorization rate of 0.1 mg/mL indigo carmine by AncCotA2 at different concentrations within 2 h.

Supplementary Table S1. Mutation site analysis of AncCotA2.

Reasons	Positions	Number
**Difficult to explain**	T2N/K25S/E33K/K63N/Q94H/E96Q/D113P/H175R/D187G/A211G/F292I/E312Q/Q345G/E348T/Q362T/H363S/T400A/S448V/T467V/H512Q/K513Q	21
**Conservative replacement**	D14E/I84V/D92E/S124T/K125R/V169A/I200L/E213D/S218T/E231D/V239A/L243M/D272E/N290Q/I302V/T307S/Y309F/I334V/Y358L/V403L/Q442N/S444T/L447I/L475I/I508V	25
**Core packing**	A171M/S186A/L194M/T196M/V254I/I255L/T300F	7
**Formed a new hydrogen bond**	A317T/A344K/T36Y	3
**Improved surface polarity/charge**	I438T/A9E/Q20K//Q38K/N75D/S78D//T79K/Q129E/P177K/A356S/A375T/A439E/P511K/	13
